# Retinal vascular assessment in psoriatic patients with and without metabolic syndrome using optical coherence tomography angiography

**DOI:** 10.1038/s41598-022-20307-3

**Published:** 2022-10-06

**Authors:** Doaa Ahmed Tolba, Rana Hussein Amin, Aya Magdi Alorbani, Sara Mamdouh Esmat

**Affiliations:** 1 Faculty of Medicine, Ophthalmology Department, Cairo University, Kasr Alainy Street, Cairo, 11956 Egypt; 2 Faculty of Medicine, Dermatology Department, Cairo University, Cairo, Egypt

**Keywords:** Health care, Medical research, Risk factors

## Abstract

To evaluate the retinal vasculature in psoriasis patients and detect if metabolic syndrome is an additional risk factor. This cross-sectional analytic study was carried out on 80 eyes of 80 subjects; 28 eyes with psoriasis only (PS group), 12 eyes with additional metabolic syndrome to psoriasis (PMS group) and 40 eyes healthy controls (HS). The retinal capillary plexuses were evaluated by OCTA. The disease activity was evaluated by the Psoriasis Area and Severity Index (PASI) score and extent. The superficial capillary plexus (SCP) vascular density was significantly lower in PS group than HS while in PMS it was significantly lower only in whole image and superior and temporal perifoveal areas (*p*-value = 0.020, 0.030, 0.001 respectively). The changes correlated with the disease duration. The vascular density of the deep capillary plexus (DCP) was significantly lower in both PS and PMS groups (*p*-value < 0.001). Psoriatic patients are at a higher risk of developing retinal vascular complications even without evident clinical ocular disease. It was noted that the presence of metabolic syndrome contributes as an additional risk factor in possible visual loss secondary to ischemic changes that are likely to start in the DCP and progress to involve all levels.

## Introduction

Psoriasis approximately affects 2–3% of people worldwide^1^ with a reported prevalence of psoriasis in Egypt ranging from 0.19 to 3%^2^. Even though it can present at any age, there is a bimodal peak at the ages between 20–30 years and 50–60 years old^3^.

Psoriasis, as a chronic inflammatory skin disorder, is characterized by a variety of immunologic and inflammatory changes^4^. It was proven to be linked to metabolic syndrome (MS) and so predisposes to atherogenesis, peripheral insulin resistance, development of arterial hypertension, type II diabetes mellitus and endothelial dysfunction^5–9^. Psoriasis is directly linked to vascular endothelial growth factors (VEGF) which explains why one of the initial events in the disease development is angiogenesis occurring even before plaque formation. The disease also affects the VEGF high-affinity receptors expression in keratinocytes and endothelial cells in the papillary dermis of sick patients^10^.

Regarding ocular manifestations of psoriasis, the latter is a well-recognized cause for anterior and posterior uveitis^11,12^, dry eye and blepharitis in patients with psoriatic arthritis^13^. Due to the close association of psoriasis to MS and increased cardiovascular abnormalities^5–9^, more focus has been turned to the changes that happen in the retinal and choroidal circulations in psoriatic patients^14,15^. A study reported that severe psoriasis appears to be related to increased subfoveal choroidal thickness (SFCT) as a consequence of possible inflammatory cascades that attribute to the pathogenesis of the disease^14^. Psoriasis itself can affect ganglion cell complex thickness and MS may cause additional damage to the retina and macula in psoriasis patients as reported by Selma et al.^15^.

Therefore, further investigations are required to study the effect of psoriasis and its inflammatory pathogenesis on the posterior segment of the eye. To our knowledge, no previous study investigated the effect of psoriasis with and without MS on the retinal vasculature using optical coherence tomography angiography (OCT-A).

## Methods

This is a cross-sectional case–control study in which 80 eyes of 80 subjects were evaluated. Forty subjects had non-ocular dermatologic psoriasis; 28 patients with psoriasis only (PS) while 12 patients had metabolic syndrome (PMS) in addition to psoriasis. Forty sex and age-matched healthy volunteers were randomly recruited as a control group (HS). Psoriasis patients were recruited from Kasr Al-Ainy Psoriasis Unit, Cairo University.

Written informed consents were taken from each participant before recruitment, and the approval of the scientific committee of the ophthalmology department in Cairo University was obtained (5/7/2020-1) and followed the tents of the declaration of Helsinki. Pregnant and lactating females, patients with erythrodermic or pustular psoriasis, or on systemic treatment as acitretin were excluded. Patients and controls with present or past history of autoimmune disorders, malignancy, or other major systemic diseases (e.g. renal, liver or cardiac disease) were excluded. Patients were subjected to full history taking and a complete dermatological examination. The extent of disease using the rule of nine^16^ was determined, as well as disease severity using Psoriasis Area and Severity Index (PASI) score^17^. Blood pressure was recorded as the average of 2 measurements after subjects had been sitting for five minutes. Waist circumference was measured (using a measuring tape) in a horizontal plane midway between the lowest rib and the iliac crest to the nearest 0.1 cm at the end of normal expiration^18^. Patients underwent laboratory investigations to measure fasting blood glucose, triglyceride, and high-density lipoprotein levels. According to the American Heart Association^19^, an individual is diagnosed with MS if they fulfill three out of five criteria: (1) Waist circumference ≥ 102 cm in men, ≥ 88 cm in women, (2) Fasting blood sugar ≥ 100 mg/dL, (3) Blood pressure ≥ 130/85 mm Hg, (4) Triglycerides ≥ 150 mg/dL and (5) High-density lipoprotein < 40 mg/dL in men and < 50 mg/dL in women.

During ophthalmological examination, patients with associated uveitis (by history or discovered by examination), glaucoma, high refractive errors (myopia > 6 D and hyperopia > 3 D), diabetic or hypertensive retinopathies, age-related macular degenerations, cataract or other media opacities and previous ocular surgeries were excluded. Assessment included full history taking, best corrected visual acuity measurement (BCVA), slit lamp examination, fundus examination, and intraocular pressure (IOP) measurement by Goldmann applanation tonometry. Retinal OCT-A assessment was performed using OptovueAngioVue® (Optovue, Inc., Freemont, CA), which uses a split-spectrum amplitude-decorrelation angiography algorithm to minimize motion noise. This system also allows quantitative analysis since it provides numerical data about flow area and flow density maps. The scanning area was captured in 6 × 6 mm sections centered on the fovea without the need for manual corrections. At each location, two consecutive B-scans were captured each containing 304 A-scans (304 B-scan locations 9.9 μm apart). It then shows 4 slabs: “superficial” and “deep” inner retinal capillary vascular plexuses (SCP and DCP), outer retina, and choriocapillaris. The en-face images of SCP were obtained with a slab between an inner boundary at 5.6 µ beneath the inner limiting membrane (ILM) and an outer boundary at 12.6 µ beneath the inner plexiform layer. The en-face images of DCP were obtained with a slab between the inner and outer boundaries, respectively, at 15.6 and 70.2 µ beneath the inner plexiform layer. The following parameters were assessed by OCT-A:

### Qualitative analysis

(1) Perifoveal anastomotic capillary arcade disruption in the SCP (when extending over 1 quadrant of the entire length). (2) Areas of capillary non-perfusion/ hypo perfusion (presenting as irregular hypo intense grayish areas. (3) Disorganization of the superficial and deep capillary network (defined as localized or diffuse loss of the normal architecture of capillary network). (4) Intra-retinal cystoid spaces (presenting as well-defined black roundish areas without any signal on OCT-A)^20^. The qualitative analyses of OCT-A being subjective were performed by two independent readers.

### Quantitative analysis

The foveal avascular zone (FAZ) area and capillary vessel density (CVD) measurements were performed using ImageJ software and were converted into black and white images using the ImageJ software.

1. *The FAZ area* was measured in square millimeters automatically using the software. The FAZ region was defined as a predetermined area with absolute no flow, and the average of the FAZ area was calculated throughout the thickness of the inner two-thirds of the fovea.
2. *The CVD* following binary reconstruction of images, was defined as the percentage of the sample area occupied by vascular lumens. AngioAnalytics software (version 2015.100.0.35) was used. It calculates the relative density of flow as a percentage of the entire examined area. CVD in the SCP and DCP are displayed as a percentage numerically in tables and qualitatively in color coded vessel density maps; where the areas of severe ischemia are color coded as dark blue.

An *OCT-A en face image* then reveals the percentage of pixels of vessels in the studied sectors or in the whole en face image based on the binary image. The percentage of vessels was defined in the following zones: (1) Whole image, (2) Para-fovea (surrounding the fovea up to 3 mm), and (3) Peri-fovea (surrounding the para-fovea up to 6 mm).

Outcome parameters:

1. To detect any abnormalities in superficial or deep retinal capillary plexuses in psoriasis patients.
2. To detect if MS associated with psoriasis is an additional risk factor.
3. Correlation between any OCT-A changes and psoriasis disease activity score, extent, and duration.

### Statistical methods

Data were analyzed using SPSS win statistical package version 26. The normality of the data was tested using the Kolmogorov–Smirnov test. Numerical data were summarized as means and standard deviations (SD) or medians and ranges as appropriate. Medians were used mainly for skewedness and not normally distributed data. While qualitative data were described as Frequencies and percentages. Comparison between more than two groups for numerical variables was done using ANOVA. Post hoc multiple comparisons were made using the Tukey test. Relation between qualitative data was done using Chi-square test or Fisher’s exact test as appropriate. Pearson correlation was used to correlate continuous data. A (*p* ≤ 0.05) was considered significant.

## Results

This study included 28 eyes of 28 patients with psoriasis only (PS group), 12 eyes of 12 patients with additional metabolic syndrome to psoriasis (PMS group) and 40 eyes of 40 healthy controls (HS group). All groups were age and sex matched. All demographic and clinical details are shown in Table 1. No statistically significant differences in IOP or refraction were found between patients and controls. All subjects had clinically normal fundus examination. The median duration of the disease was 10 years (IQR = 1–35) in PS group and 10 years (IQR = 3–10) in PMS group. The median PASI score was 13.6 (IQR = 0.4–33.4) in PS group and 10.0 (IQR = 8.5–10.2) in PMS group while the median disease extent was 30 (IQR = 25–90) in PS group and 25 (IQR = 20–50) in PMS group. No significant differences in PASI scores, disease extent or disease duration were found between PS and PMS groups.

**Table 1:** Demographic and clinical data of psoriasis patients, metabolic syndrome and controls.

Characteristics	PS	PMS	HS	p-value
**Age**				
Mean ± SD	41.4 ± 16.1	43.0 ± 9.8	35.8 ± 10.7	0.330
**Sex**				
Female	6 (21.4%)	2 (16.7%)	22 (55%)	
Male	22 (78.6%)	10 (83.3%)	18 (45%)	0.0718
**VA**				
Mean ± SD	0.9 ± 0.2	0.8 ± 0.3	0.9 ± 0.1	0.614
**IOP**				
Mean ± SD	15.0 ± 2.7	13.5 ± 2.5	15.9 ± 1.9	0.085
**Disease duration**				
Median (range)	10 (1–35)	10 (3–10)		0.442

*PS* psoraisis group, *PMS* psoraisis and metabolic syndrome, *HS* healthy control, *VA* visual acuity, *IOP* intraocular pressure.

### Analysis of OCT-A findings

#### Foveal avascular zone (FAZ) and foveal vascular density (FD)

**Figure 1:**
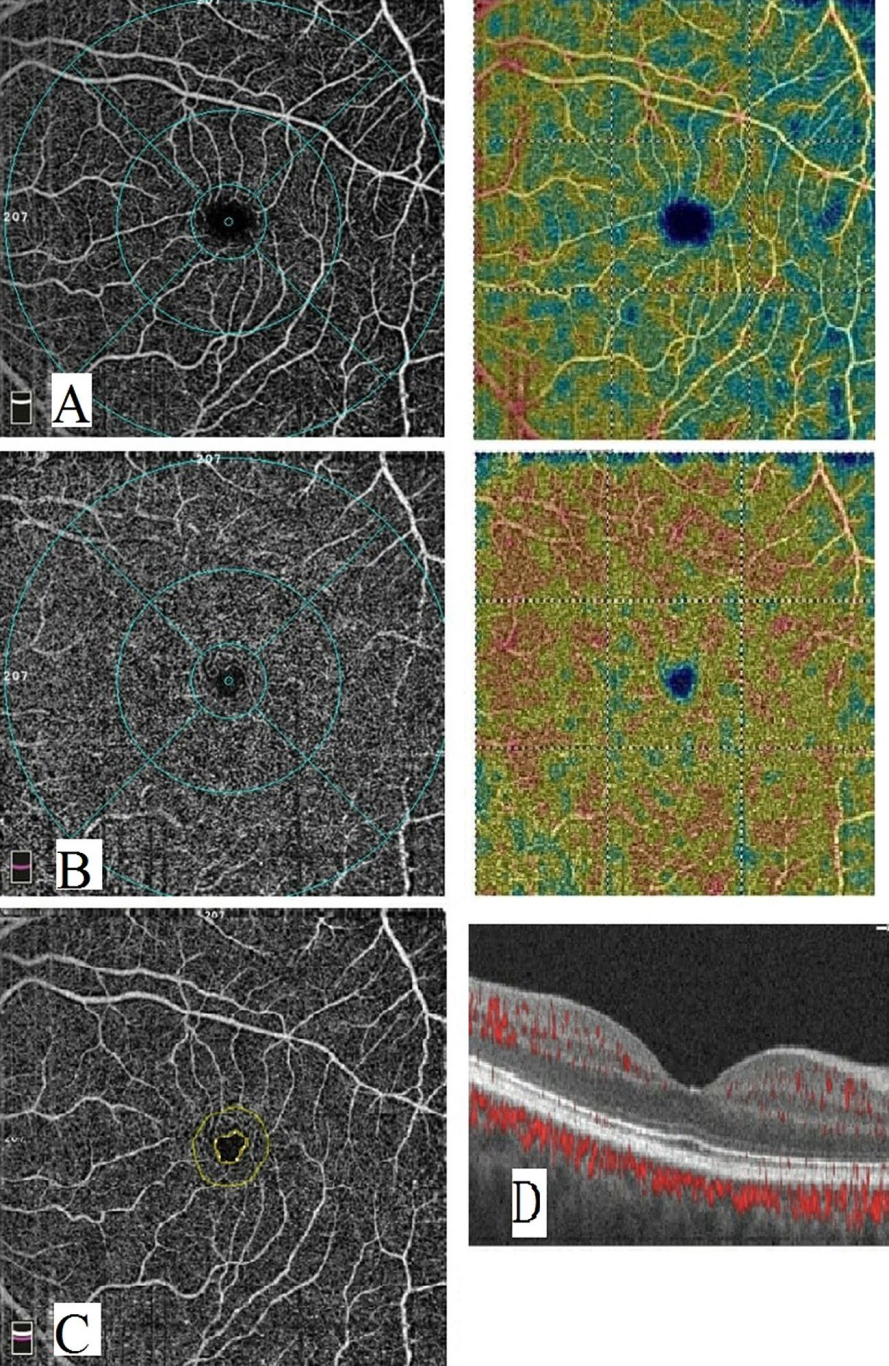
Optical coherence tomography angiography image of a normal subject. (**A**) enface image at the level of SCP. (**B**) enface image at the level of DCP. (**C**) FAZ. (**D**) B scan image showing blood flow at the level of SCP, DCP and choriocapillaris.

**Figure 2:**
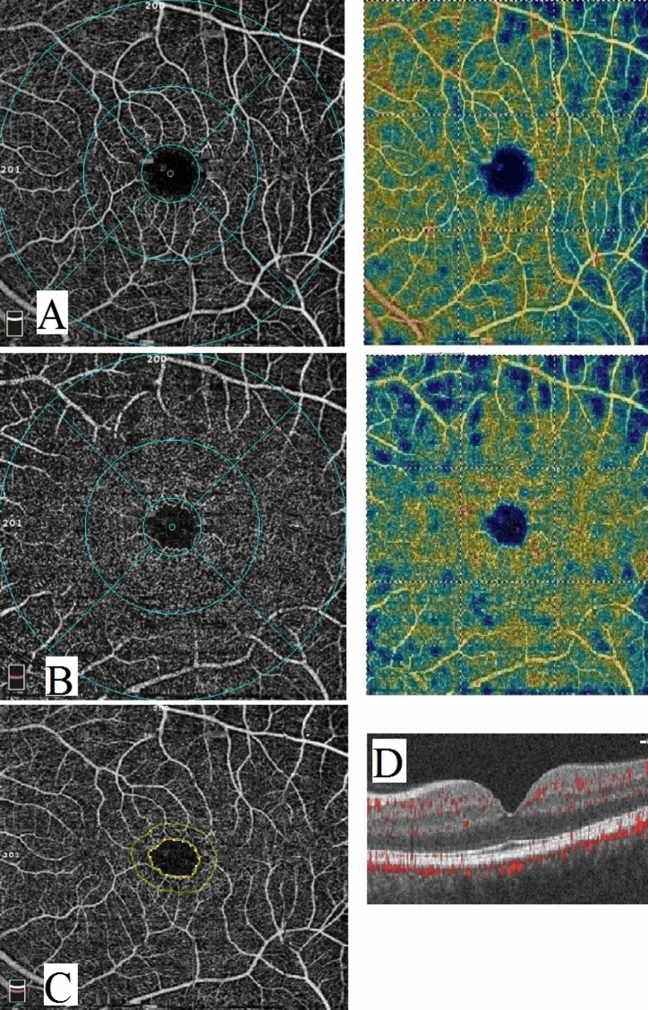
Optical coherence tomography angiography image of a psoriasis patient. (**A**) enface image at the level of SCP. (**B**) enface image at the level of DCP. (**C**) FAZ. (**D**) B scan image showing blood flow at the level of SCP, DCP and choriocapillaris.

**Figure 3:**
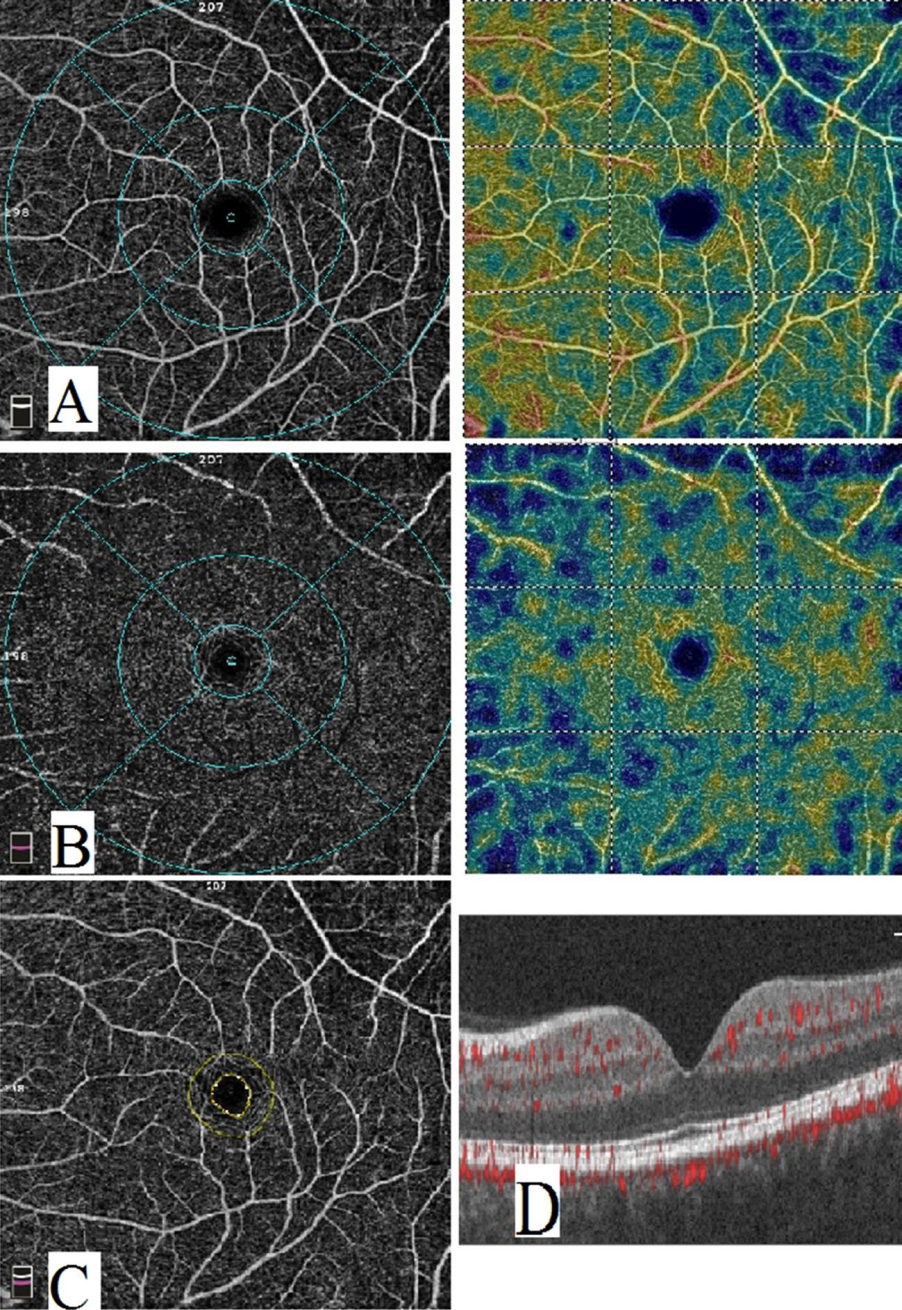
Optical coherence tomography angiography image of a psoriasis patient with metabolic syndrome. (**A**) enface image at the level of SCP. (**B**) enface image at the level of DCP. (**C**) FAZ. (**D**) B scan image showing blood flow at the level of SCP, DCP and choriocapillaris.

The differences in FAZ area between all groups were not statistically significant. On the other hand, the foveal vascular densities (FD) were significantly lower in both PS and PMS groups than in HS group (*p*-value = 0.003, 0.052 respectively) while there was no significant difference in the foveal density between PS and PMS groups (*p*-value = 0.754) as shown in Table 2 and Figs. 1, 2, and 3. Central foveal thickness (CFT) was significantly higher in the PMS group when compared with PS group and controls (*p*-value < 0.001).

**Table 2:** Comparison of FAZ, FD and CFT between groups.

Characteristics	PS	PMS	HS	p-value			
	Mean ± SD	Mean ± SD	Mean ± SD	All groups	PS/HS	PMS/HS	PMS/PS
FAZ	0.299 ± 0.072^a^	0.280 ± 0.045^a^	0.283 ± 0.088^a^	0.660	0.406	0.925	0.494
FD	53.0 ± 4.1^a^	53.4 ± 4.2^a^	55.8 ± 3.2^b^	0.007	0.003	0.052	0.754
CFT	241.9 ± 20.1^a^	270.3 ± 11.9	246.4 ± 15^a^	< 0.001	0.275	< 0.001	< 0.001

*PS* psoraisis group, *PMS* psoraisis and metabolic syndrome, *HS* healthy control, *FAZ* foveal avascular zone, *FD* foveal density, *CFT* central foveal thickness. There was a statistically significant difference between a variable with a different letter, no significant difference between a variable with the same letter.

#### Measurements of superficial and deep capillary plexus

Regarding the SCP, the vascular density in all measurements was significantly lower in PS group than in control group while in PMS it was significantly lower only in whole image and superior and temporal perifoveal areas (*p*-value = 0.020, 0.030, 0.001 respectively). However, no significant difference was found between PS and PMS groups as shown in Table 3 and Figs. 1, 2, and 3 respectively.

**Table 3:** Comparison of quantitative OCTA measurements between psoriasis group, metabolic syndrome and controls.

Characteristics	PS	PMS	HS	p-value			
	Mean ± SD	Mean ± SD	Mean ± SD	All groups	PS/HS	PMS/HS	PMS/PS
Superficial Wi VD	49.6 ± 3.7^a^	49.5 ± 2.2^a^	51.7 ± 2.3^b^	0.005	0.004	0.020	0.903
Superficial parafoveal VD	51.7 ± 5.4^a^	52.9 ± 2.2	54.4 ± 2.6^b^	0.021	0.006	0.232	0.385
Superficial perifoveal VD (temporal)	46.8 ± 4.4^a^	46.2 ± 2.1^a^	48.6 ± 2.6^b^	0.030	0.033	0.030	0.580
Superficial perifoveal VD (superior)	49.5 ± 3.8^a^	48.2 ± 3.4^a^	51.9 ± 2.5^b^	< 0.001	0.003	0.001	0.253
Superficial perifoveal VD (nasal)	53.8 ± 3,6^a^	54.7 ± 1.9^a^	55.7 ± 2.3^b^	0.023	0.006	0.256	0.367
Superficial perifoveal VD (inferior)	50.6 ± 3.9^a^	50.8 ± 2.6	52.2 ± 3.0^b^	0.104	0.046	0.189	0.854
Deep Wi VD	49.9 ± 5.4^a^	45.7 ± 4.1^b^	56.20 ± 3.8^c^	< 0.001	< 0.001	< 0.001	0.007
Deep parafoveal VD	53.8 ± 4.3^a^	48.8 ± 2.8^b^	59.1 ± 2.9^c^	< 0.001	< 0.001	< 0.001	< 0.001
Deep perifoveal VD (temporal)	52.9 ± 5.7^a^	49.7 ± 2.8^b^	59.5 ± 2.6^c^	< 0.001	< 0.001	< 0.001	0.023
Deep perifoveal VD (superior)	49.4 ± 6.8^a^	44.5 ± 4.2^b^	56.7 ± 5.0^c^	< 0.001	< 0.001	< 0.001	0.015
Deep perifoveal VD (nasal)	50.7 ± 6.5^a^	46.9 ± 6.5^a^	57.8 ± 4.3^b^	< 0.001	< 0.001	< 0.001	0.053
Deep perifoveal VD (inferior)	50.2 ± 7.8^a^	47.7 ± 4.7^a^	58.4 ± 3.7^b^	< 0.001	< 0.001	< 0.001	0.166

*PS* psoraisis group, *PMS* psoraisis and metabolic syndrome, *HS* healthy control, *Wi* Whole image, *VD* Vessel density. There was a statistically significant difference between a variable with a different letter, no significant difference between a variable with the same letter.

On the other hand, all measurements of the vascular density in the DCP were significantly lower in both PS and PMS groups compared to the controls (*p*-value < 0.001). Moreover, the DCP vascular density was significantly lower in PMS group than in PS in all measurements except the inferior perifoveal area as shown in Table 3 and Figs. 1, 2, and 3 respectively.

#### Qualitative parameters in superficial and deep capillary plexus

Areas of capillary non-perfusion in the SCP were found in 6 eyes of PS group and 4 eyes of PMS group versus 4 eyes in control group while the DCP showed areas of capillary non-perfusion in 7 eyes of PS group and 4 eyes of PMS group versus no eyes in control group. No disorganization of capillaries or intra-retinal cystoid spaces or disruption of the parafoveal arcades were detected at all.

#### OCTA findings and parameters of the disease

Pearson correlation coefficients of the SCP vascular density with the psoriasis disease duration showed a statistically significant negative correlation (r = − 0.542 P value < 0.001). On the other hand, we found no correlation with the disease extent and PASI score.

DCP vascular density had no significant correlation with any of the disease duration, extent or PASI score (*p*-value = 0.77, 0.964 and 0.922 respectively).

## Discussion

Both, psoriasis and MS, have abnormal expression of inflammatory and anti-inflammatory markers that can result in increased oxidative damage to nucleic acids^21^. Disturbances in levels of adipokines (e.g. decreased levels of adiponectin and omentin and increased levels of leptin and resistin) may play a role in increasing the prevalence of cardiovascular disease in psoriasis patients^22^. That is why, for the past decade, researchers have started paying more attention to the vascular anomalies occurring in the posterior segment of such patients even in the absence of clinically evident ocular disease.

To date, this is the first time to study the effect of psoriasis with and without metabolic syndrome on retinal microcirculation compared to healthy controls using OCT-A. Our results showed that all the values of FD, SCP and DCP in both psoriasis groups were significantly lower compared to healthy subjects. This agrees with the results published by Castellino N et al. in 2021 which found significantly lower vascular densities in both, superficial and deep plexuses, in psoriatic patients when compared to controls^23^. Yet, it seems that the presence of MS adds an additional ischemic risk in decreasing the values of some areas of the DCP when compared to DCP of psoriatic patients without MS. Our PMS group had significantly lower DCP values compared to PS group in all quadrants except for nasal and inferior perifoveal quadrants. There are no reports in the literature comparing OCT-A values in MS compared to normal individuals yet values for SCP and DCP were definitely lower in diabetics^24^ and hypertensive patients^25^ without evident clinical diabetic nor hypertensive retinopathy respectively. Interestingly, a recent study on the effect of MS on retinal thickness in psoriasis patients has shown that MS resulted in a significant decrease in superior retinal nerve fiber layer thickness compared to psoriasis patients without MS^15^. These results seem to point towards a conclusion that retinal ischemic changes occur in MS patients necessitating close follow-up ocular exams and more frequent testing. SCP affection was more evident in PS than PMS which emphasis the possibility of retinal vascular involvement in psoriasis patients even in the absence of MS as found by Castellino et al.^23^.

It is not known whether DCP measurements are more sensitive to systemic ischemic changes than SCP or not. Arfeen et al. studied OCT-A measurements in systemic lupus erythematosus (SLE) patients without evident ocular disease compared to normal individuals. They concluded that all of DCP quadrants showed statistically significantly lower values compared to normal subjects while most of SCP values showed no differences^26^. In another study on Behcet’s disease, areas of hypoperfusion in SCP were smaller than areas of non-perfusion in DCP^27^. In spite of these conclusions, it would have been expected to find a significant correlation between affection of DCP and disease duration and extent (PASI score); yet this was not the case. A significant negative correlation between SCP values and disease duration was found instead. Perhaps this dilemma could be elucidated better in future work with recruitment of larger number of patients and categorizing the patients into groups according to severity of disease. Another possible explanation could be the early affection of the DCP independent on the duration of the disease.

The lack of significance between the results of FAZ of the 3 groups agrees with the results published by several authors when comparing patients having systemic ischemic illness with controls whether in psoriasis^3^ or other diseases such as SLE^26^ and Behcet’s disease^27^. This points to the superior value and higher sensitivity of OCT-A as a screening tool compared to fundus fluorescein angiography (FFA) in the detection of subclinical degrees of ischemia by analyzing accurately the areas of superficial and deep capillary plexuses. Considering that changes tend to occur in the deep and superficial plexuses first, OCT-A should be used as an additional tool to FFA and optical coherence tomography (OCT) in the assessment of unexplained visual loss in ischemic patients without clinically evident disease.

Similar to previous reports on the effect of systemic ischemia and/or inflammation on the retinal microcirculation^23–26^, we conclude that psoriatic patients are at a higher risk of developing retinal vascular complications secondary to decreased perfusion even in cases without evident clinical ocular disease. It should be noted that the presence of MS contributes as an additional risk factor in possible visual loss secondary to ischemic changes that are likely to start in the DCP and progress to involve all levels at later stages. Therefore, OCT-A can act as a valuable screening tool in patients with systemic disease with high prevalence of cardiovascular morbidity.

## Data Availability

The datasets used and/or analyzed during the current study available from the corresponding author on reasonable request.
